# Maf-family bZIP transcription factor NRL interacts with RNA-binding proteins and R-loops in retinal photoreceptors

**DOI:** 10.1101/2024.09.19.613899

**Published:** 2024-09-19

**Authors:** Ximena Corso-Díaz, Xulong Liang, Kiam Preston, Bilguun Tegshee, Milton A. English, Jacob Nellissery, Sharda Prasad Yadav, Claire Marchal, Anand Swaroop

**Affiliations:** 1 Neurobiology, Neurodegeneration and Repair Laboratory, National Eye Institute, National Institutes of Health, MSC0610, 6 Center Drive, Bethesda, Maryland, 20892 USA; 2 Department of Ophthalmology, Byers Eye Institute, Stanford University, Stanford, California, USA.; 3 In silichrom Ltd, 15 Digby road, RG14 1TS Newbury, United Kingdom

## Abstract

RNA-binding proteins (RBPs) perform diverse functions including the regulation of chromatin dynamics and the coupling of transcription with RNA processing. However, our understanding of their actions in mammalian neurons remains limited. Using affinity purification, yeast-two-hybrid and proximity ligation assays, we identified interactions of multiple RBPs with NRL, a Maf-family bZIP transcription factor critical for retinal rod photoreceptor development and function. In addition to splicing, many NRL-interacting RBPs are associated with R-loops, which form during transcription and increase during photoreceptor maturation. Focusing on DHX9 RNA helicase, we demonstrate that its expression is modulated by NRL and that the NRL-DHX9 interaction is positively influenced by R-loops. ssDRIP-Seq analysis reveals both stranded and unstranded R-loops at distinct genomic elements, characterized by active and inactive epigenetic signatures and enriched at neuronal genes. NRL binds to both types of R-loops, suggesting an epigenetically independent function. Our findings suggest additional functions of NRL during transcription and highlight complex interactions among transcription factors, RBPs, and R-loops in regulating photoreceptor gene expression in the mammalian retina.

## Introduction

Cell type-specific and quantitatively precise decoding of genetic information produces a plethora of divergent cellular morphologies and functions during development^1–3^. The initiation of DNA-dependent RNA synthesis by RNA Polymerase II is coordinated by complex interactions among chromatin modifying proteins, ubiquitous and cell-type specific transcription factors, and transcriptional machineries^4–6^. Combinatorial and sequence-specific binding of trans-acting regulatory proteins to *cis*-regulatory elements including enhancers and promoters is facilitated by specific genome topologies, resulting in productive transcriptional output by assembling phase-separated condensates^4,6^. Concurrent control mechanisms meticulously guide every step of the genetic program. Curiously, cell type- and tissue-specific transcription factors are also reported to contribute to additional transcriptional and post-transcriptional regulatory steps, including 3-D chromatin dynamics and splicing^7–9^, to achieve stringent spatiotemporal control of gene expression patterns.

Gene regulatory networks underlying neuronal differentiation in the mammalian retina^10–14^, especially photoreceptors^15–17^, offer an attractive system to dissect divergent roles of transcriptional factors in controlling quantitatively precise cell type-specific gene expression. The Maf-family basic motif leucine zipper (bZIP) transcription factor NRL determines rod photoreceptor cell fate as its loss leads to retina with only cone photoreceptors^18^, whereas ectopic expression of NRL in developing cones results in generation of rods^19^. Continued expression of NRL is essential for maintaining rod function^20^, and mutations affecting NRL activity are associated with retinopathies^21–24^. NRL establishes rod cell identity and function by regulating precise transcriptional output of thousands of genes^25–27^ in collaboration with cone rod homeobox (CRX)^28–30^ and other regulatory proteins^15,16,31,32^. Given its fundamental role as a primary activator of rod expressed genes and continuous high-level expression during the life of rod photoreceptors, we hypothesized the involvement of NRL in modulating additional steps during the transcription process. This prediction was strengthened by transcriptional activation synergy observed in rhodopsin regulation between NRL and NonO/p54^nrb 33^. NonO, a ubiquitous nuclear paraspeckle protein, binds to DNA, RNA as well as proteins, and regulates distinct cellular events including the coupling of the circadian clock to cell cycle and transcription to splicing^34–36^.

Here, we focused on identifying additional roles of NRL in guiding rod gene expression by characterizing NRL-interacting proteins from the mammalian retina using multiple complementary approaches. We discovered an over-representation of RNA-binding proteins (RBPs) among NRL interactors, with almost half of high confidence interacting proteins associated with R-loops, which are DNA structures comprised of RNA-DNA hybrids and displaced single-stranded DNA. We then investigated the interaction of NRL with two key RNA helicases, DHX9 and DDX5, which mediate resolution of R-loops and further evaluated R-loop dynamics and epigenetic signatures in the retina. Our study underscores the importance of RBPs and R-loop formation as key NRL-interacting regulators of gene expression in retinal rod photoreceptors.

## Results

### RNA binding proteins constitute a large cohort among NRL interactors

**Figure 1.**
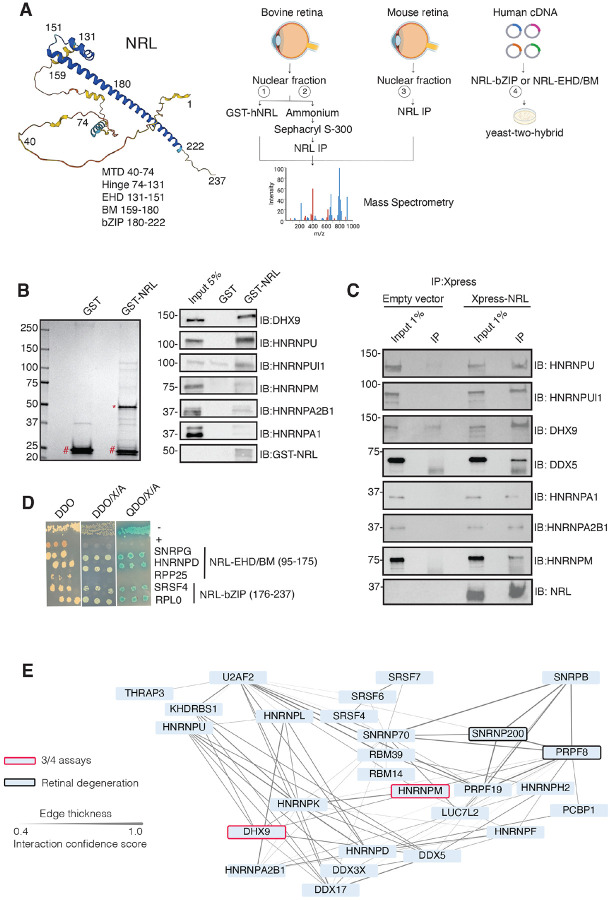
NRL interacts with RNA-binding proteins. A) Summary of experimental strategies used to identify NRL interactors. An AlphaFold-predicted of human NRL model is shown, with protein domains highlighted. MTD = minimal transactivation domain, EHD = extended homology domain, BM = basic domain, bZIP = basic leucine zipper. Four different assays were performed to identify NRL interactors. Affinity purifications with Glutathione and NRL antibodies were performed from bovine and mouse retinal lysates and subjected to mass spectrometry. Yeast-two-hybrid experiments from human retina cDNA using NRL bZIP and EHD/BM domains were also performed. Eye and plasmid depictions were obtained from BioRender.com. B) Coomassie staining showing proteins from bovine retina purified with human NRL fused to GST (GST-NRL;*). Purified GST (#) was used as control. Experiments were performed three times with different retinal lysates. Western blot of RBPs identified by LS/MS harboring >10 times enrichment in at least one GST-NRL replicate compared to controls is shown to the right. C) Western blot showing detection of different RBPs co-immunoprecipitating with NRL in HEK293 cells overexpressing Xpress-tagged NRL. Empty vector containing Xpress tag was used as control. D) Yeast colonies from yeast-two-hybrid screens showing positive interaction between RBPs and NRL extended homology domain (EHD) and basic leucine zipper (bZIP) domain. Colonies were plated against controls on SD/–Leu/–Trp (Double Dropout; DDO), SD/-Trp/-Leu/X-alpha-gal/Aureobasidin-A (DDO/X/A) and SD/-Trp/-Leu/-Ade/-His/X-alphagal/Aureobasidin-A (QDO/X/A) plates. P53 and Lamin were used as positive and negative controls, respectively. E) PPI network showing RBP experimental interactions from String. Proteins represent a subnetwork of NRL-interacting RBPs found in two out of four assays summarized in A. The edge thickness represents the confidence score with a cutoff of 0.4. Proteins identified in 3 out of 4 assays are highlighted with a red border. Proteins with known causative mutations for inherited retinal degeneration are shown with a black border.

To identify new NRL protein partners that mediate its function in rod photoreceptors, we first conducted an unbiased protein interaction screening. This involved a GST-NRL affinity purification using bovine retina nuclear extracts. This strategy was followed by co-immunoprecipitation (IP) from NRL-enriched high molecular mass fractions of bovine retina, NRL co-IP from mouse retina, and yeast-two-hybrid assays with a NRL domain bait against a human retina “prey” library (schematically shown in Fig. 1A).

We identified 28 proteins that were significantly enriched (>2-fold, *p* <0.05) compared to control samples in three independent GST-NRL pull-down experiments (Figure 1B, Table S1). Most proteins were not detected in any of the GST control samples. RBPs represented over 40% of NRL interactors in this set (12/28) (Table S1). We confirmed 6 of the strongest interactors by immunoblot analysis of GST-pulldown proteins using specific antibodies (Fig. 1B). Notably, interaction of NRL with DHX9, HNRNPU or HNRNPUl1 remained strong even after incubation of the nuclear lysate with Benzonase, a promiscuous nuclease that digests all nucleic acid species, whereas HNRNPA1 and HNRNPM were not pulled down after treatment, indicating a requirement of RNA for the latter set of interactions (Fig. S1A).

Independently and concurrently, immunoprecipitation using anti-NRL antibody of NRL-enriched high molecular mass fractions from the bovine retina, followed by mass-spectrometry analysis, identified several RBP interactors in addition to known interacting proteins such as CRX, NR2E3 and NonO/p54^nrb^ (Table S2). Prioritization of RBPs was done by selecting those with a combined PSM higher than 10 and at least two-fold enrichment. Notably, the most enriched proteins included DHX9, HRRNPU and HNRNPM. Additionally, NRL co-IP experiments using mouse retina nuclear extracts followed by mass spectrometry further validated these findings (Table S2). We also confirmed the interaction between NRL and several of the identified proteins by transfection of tagged constructs in HEK293 cells overexpressing human NRL (Fig. 1C). A limited yeast two-hybrid screening using the NRL bZIP or extended homology domain (EHD) domain baits also identified several RBPs, including SNRPG, HNRNPD, SRSF4, and RPLP0, which were confirmed by positive bait and prey interactions (blue color) (Fig. 1D).

Candidate NRL-interacting RBPs, obtained from all four assays (n = 197), were annotated with curated protein-protein interactions (PPI) obtained from String (https://string-db.org/)^37^ to generate a PPI network (Fig. S1B). This network displayed highly interconnected nodes that may indicate interactions with NRL in different cellular contexts. Notably, 30 of these proteins were identified in at least two assays, and two of these, DHX9 and HNRNPM, in three assays (Figure S1B). Of these, twenty-seven RBPs show a high degree of interaction and form a highly interconnected subnetwork (Figure 1E). Notably, two of the RBPs (PRPF8 and SNRNP200)^38^ have been implicated in photoreceptor degeneration and harbor substantial interactions in this subnetwork (Figure 1E, black rectangle). We also noted that some RBPs in the network (DHX9, DDX5, DDX17, DDX3X) are RNA helicases with prominent roles in the regulation of R-loops, which are non-B DNA structures comprising of RNA-DNA hybrids and displaced single-stranded DNA with multiple regulatory roles in transcription^39^. We then identified 67 high-confidence R-loop binding proteins from four independent studies^40–43^ by determining shared proteins in their respective R-loop proteomes. Strikingly, 50% of NRL-interacting RBPs were also identified in this high confidence R-loop protein group indicating a high enrichment of R-loop-associated RBPs among NRL interactors (Fisher exact test *p* = 2E - 13) (Figure S1C, Table S3).

### NRL interacts with the R-loop helicases DHX9 and DDX5 in rod photoreceptor nuclei

**Figure 2.**
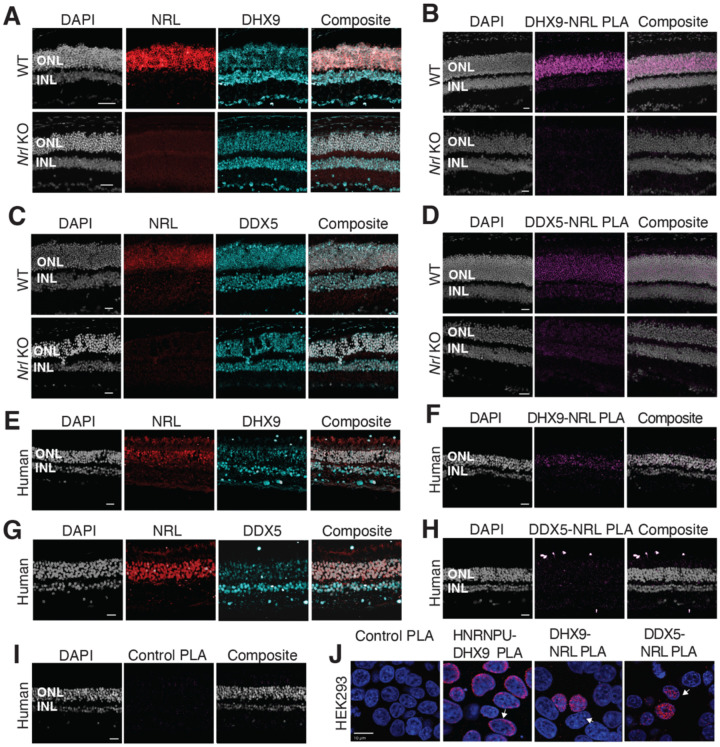
DHX9 and DDX5 are expressed in retinal photoreceptors and interact with NRL within the nuclear compartment. A) DHX9 (blue) and NRL (red) expression in adult mouse wild type (WT) and *Nrl* Knockout (KO) retina. B) Proximity ligation assay (PLA) signal (magenta) using anti-DHX9 and NRL antibodies in adult mouse WT and *Nrl* KO retina. C) DDX5 (blue) and NRL (red) expression (blue) in adult mouse WT and *Nrl* KO retina. D) PLA signal (magenta) using anti-DDX5 and NRL antibodies in adult mouse WT and *Nrl* KO retina. E) DHX9 (blue) and NRL (red) expression in adult human retina. F) PLA signal (magenta) using anti-DHX9 and NRL antibodies in the adult human retina. G) DDX5 (blue) and NRL (red) expression in adult human retina. H) PLA signal (magenta) using anti-DDX5 and NRL antibodies in the adult human retina. I) PLA signal (magenta) using no primary antibody in the adult human retina. J) PLA signal in HEK293 cells overexpressing human Xpress-NRL. DHX9 interaction with its known protein partner HNRNPU is shown in the nuclear periphery (arrow). Xpress-NRL interaction with DDX5 and DHX9 in euchromatin is shown in red (arrows). Nuclei were counterstained with DAPI (grey in human and mouse retina; blue in HEK293 cells). Scale bar = 20 μM. ONL = Outer nuclear layer; INL = Inner nuclear layer.

We further focused on the interaction of NRL with R-loop resolvases DHX9 (identified in all affinity purifications) and DDX5 (identified in endogenous NRL-pull downs from bovine and mouse retinas). DHX9 and DDX5 are shown to be present in the same protein complexes^44^. Notably, immunofluorescence of mouse retina reveals the localization of DHX9 and DDX5 in the rod nuclear periphery, which corresponds to the euchromatin compartment of murine rods (Fig. S2A). We therefore studied DHX9 and DDX5 interaction with NRL *in situ* using a proximity ligation assay (PLA). In mouse retina, DHX9 is observed in all nuclei (Fig. 2A). PLAs using anti-NRL and DHX9 antibodies show strong positive interaction signal in the outer nuclear layer (Fig. 2B) where NRL is normally expressed (Fig.2A). In contrast, no signal above background is detectable when PLA is performed in *Nrl* KO retina (Fig. 2B), suggesting the specificity of PLA signals, even though DHX9 is still expressed throughout the KO retina nuclei (Fig. 2A). DDX5 also shows widespread expression throughout the retina (Fig. 2C) but displays a lower yet specific interaction signal with NRL in the outer nuclear layer by PLA (Fig. 2D). This signal is absent in *Nrl* KO retina (Fig. 2D). Similar to mouse retina, DHX9 and DDX5 are detected in all nuclear layers of the human retina whereas NRL is present only in the outer nuclear layer of photoreceptors (Fig. 2E,G). Consistent with the results in mouse, NRL-DHX9 PLA signal is clearly detected in the outer nuclear layer of the human retina (Fig. 2F), indicating a robust and reproducible interaction between NRL and DHX9. However, NRL-DDX5 PLA signals do not seem to be above negative control (Fig. 2H, I). Interestingly, in HEK293 cells overexpressing human NRL, PLA signals are strong for both DHX9 and DDX5 throughout the nuclear compartment (Fig. 2J). DHX9 and HNRNPU serve as a positive control for PLA and are enriched mostly in the nuclear periphery (Fig. 2J). Taken together, these findings suggest a strong interaction between NRL and DHX9 throughout the nuclear compartment and a weaker or more regulated interaction of NRL and DDX5 in the retina.

### NRL regulates *DHX9* gene expression

**Figure 3.**
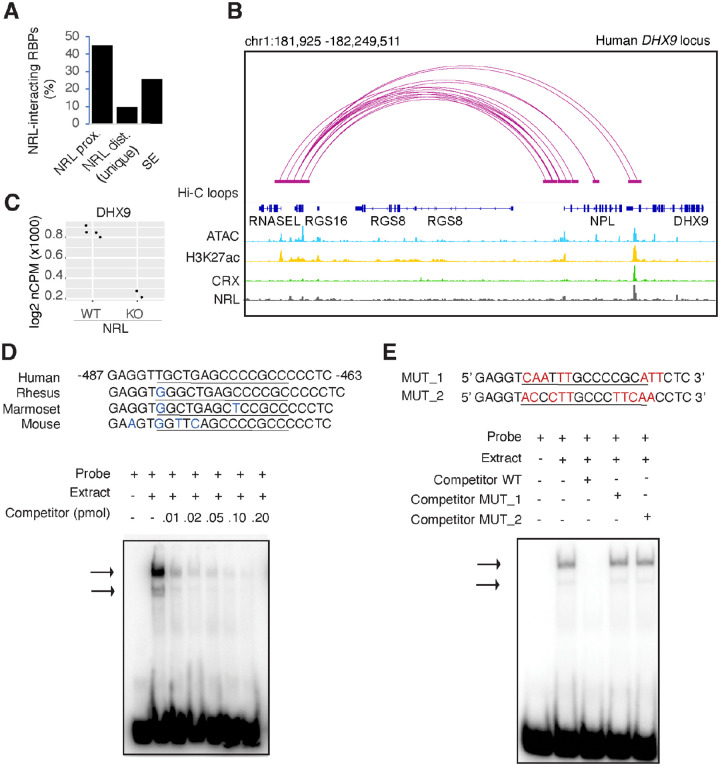
NRL genetically interacts with RBPs. A) Bar graph showing the fraction of NRL-interacting RBPs that harbor NRL proximal (< +/− 1Kb gene body) or distal (>1 Kb gene body) ChIP-Seq peaks and/or super-enhancers (SE) in the human retina (Data from *Marchal et al. 2022*). B) Genomic view of the human *DHX9* locus showing Hi-C loops, ATAC-Seq, H3K27ac, CRX-ChIP-Seq and NRL-ChIP-Seq tracks (Obtained from *Marchal et al. 2022*). C) Expression levels of *Dhx9* in wild-type and *Nrl* knockout flow-sorted photoreceptors (obtained from *Kim et al., 2016*). D) EMSA autoradiography using a probe containing an NRL motif identified at NRL-ChIP-Seq peak on the human DHX9 promoter. Specific bands (arrows) form after incubation with bovine nuclear retina extracts. The sequence of the ^32^P-labeled probe containing human NRL motif (underlined) and its homologous sequence in other mammals is shown on the top panel (blue letters indicate nucleotide differences). Competition assays were performed using unlabeled probes at increasing concentrations (pmol) as shown. E) EMSA autoradiography showing competition assays with 0.2 pmol WT and mutant DHX9 probes, MUT_1 and MUT_2 (sequences are shown in top panel; nucleotide changes are shown in red).

As transcription factors often operate on positive feedback loops, we studied whether the gene expression of NRL interactors was influenced by NRL itself. We examined the regulation of NRL-interacting RBPs by inspecting the published NRL ChIP-Seq and super enhancer profiles from the human retina^45^. Interestingly, over 50% of RBP genes possess NRL ChIP-Seq peaks and almost 25% harbor super enhancers (SE) (Fig. 3A). Fig. S3A shows a few examples of NRL binding to super enhancers regions at *DDX5*, *HNRNPU*, *DDX3X* and *THRAP3* genes. We then focused on the *DHX9* locus and could identify NRL binding peaks in both human and mouse promoter regions (Fig. 3B, Fig. S3B). In concordance, we observe lower levels of *Dhx9* transcripts in *Nrl* KO photoreceptors^26^ (Fig. 3C). Using a probe containing NRL binding motif within the human *DHX9* promoter, we demonstrate binding of bovine retina nuclear proteins by Electrophoretic Mobility Shift Assays (EMSA) (Fig. 3D). This binding is not detected by mutations in and around the putative NRL-binding site (Fig. 3D, E). Taken together, our data suggest that NRL can bind to and influence the expression of its RBP interactors.

### Interaction between NRL and DHX9 is regulated by R-loops

**Figure 4.**
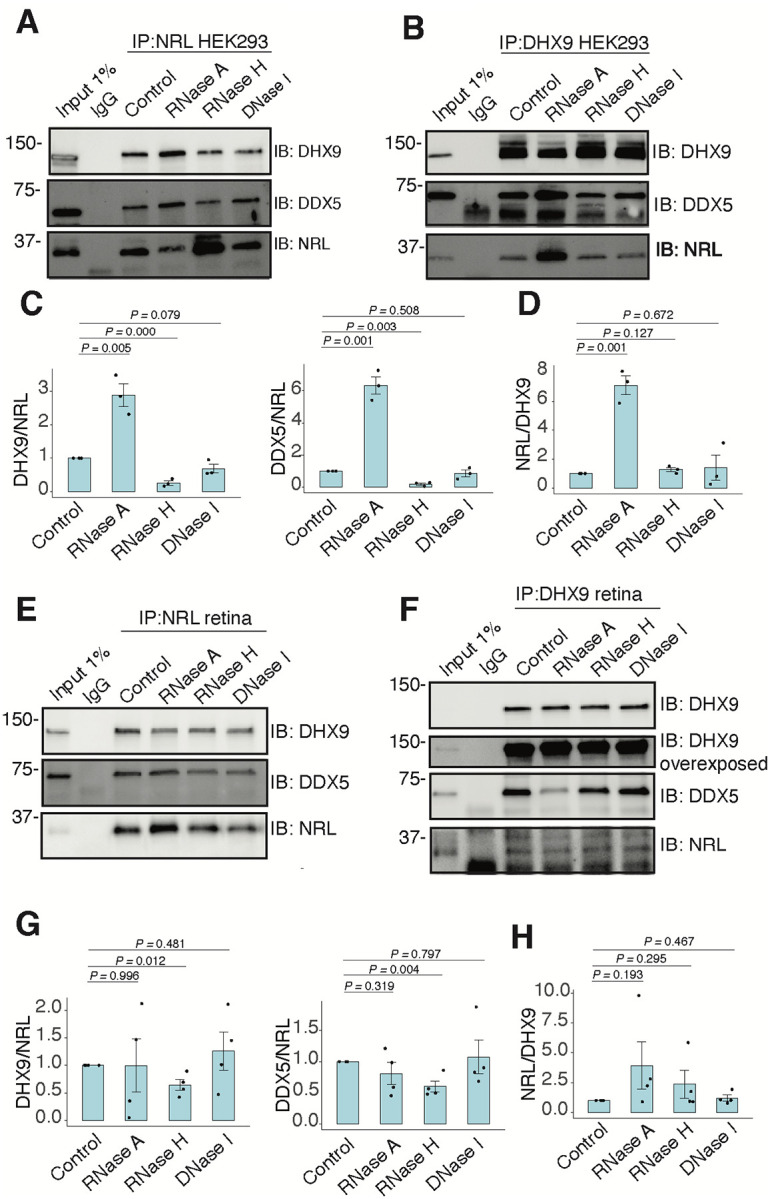
RNA:DNA hybrids regulate the interaction between NRL and DDX5/DHX9. A-B) Co-immunoprecipitation (co-IP) of DDX5 and DHX9 from HEK293 cells overexpressing NRL. Lysates were treated for 30 min with different nucleases (as shown) before incubations with respective antibodies. Immunoprecipitation (IP) of NRL (A) or DHX9 (B) and immunoblot (IB) staining for NRL, DHX9 and DDX5 is shown. C-D) Quantification of signal intensities normalized to precipitated NRL and DHX9 (shown in A and B, respectively), (n = 3). Data are presented as the mean ± SEM. An unpaired two-tailed t-test was performed to compare the means of samples against controls. E-F) Co-IP of DDX5 and DHX9 from nuclear fractions of bovine retinas. Lysates were treated for 30 min with different nucleases (as shown) before incubations with respective antibodies. Immunoprecipitation (IP) of NRL (E) or DHX9 (F) and immunoblot (IB) staining for NRL, DHX9 and DDX5 is shown. G-H) Quantification of signal intensities normalized to precipitated NRL and DHX9 (shown in E and F, respectively), (n = 4). Data are presented as the mean ± SEM. Unpaired two-tailed t test was performed to compare means of samples against controls.

We further characterized whether the interactions between NRL and DHX9 or DDX5 are regulated by different types of nucleic acids in HEK293 or bovine retina. We performed co-IP with antibodies against NRL, DHX9 and DDX5. Notably, IP using a DDX5 antibody failed to consistently pull down NRL in HEK293 cells and bovine retina, indicating that only a small pool of NRL may be involved in this interaction. In HEK293 cells overexpressing NRL, the interaction of DHX9 and NRL is enhanced upon treatment of lysates with RNase A (Fig. 4A, C), suggesting a negative regulation by RNA. This was also the case in reciprocal pull downs with DHX9 (Fig. 4B, D). On the other hand, NRL interaction with both DHX9 and DDX5 was positively regulated by DNA:RNA hybrids as it was reduced upon treatment with RNase H, which digests RNA only in the hybrid context (Fig. 4A, C). Notably, this was not observed in reciprocal pull downs with DHX9 (Fig. 4B, D). In addition, there was no influence of DNA on NRL interactions as evidenced by treatments of lysates with DNase I, which has only 1% activity on DNA:RNA hybrids (Fig. 4A–D). In contrast, pull down of DHX9 and DDX5 with endogenous NRL from bovine retina was not influenced by RNase A treatment (Fig. 4E, G). However, NRL interactions decreased upon removal of DNA:RNA hybrids (Fig. 4E, G), suggesting a role of R-loops in regulating NRL interactions. As in case of HEK293 cells, decreasing DNA:RNA hybrids did not have an effect on the amount of NRL pulled down with DHX9 (Fig. 4D, F). In contrast to NRL interactions, the binding between DHX9 and DDX5 was decreased in the bovine retina when RNase A was present (Fig. 4F and S4A), indicating a positive regulatory role of RNA in this tissue. Yet, similar to NRL interactions, the binding between DHX9 and DDX5 was reduced by RNase H treatment in HEK293 cells, but not in the bovine retina (Fig. 4B, F and S4B).

**Figure 5.**
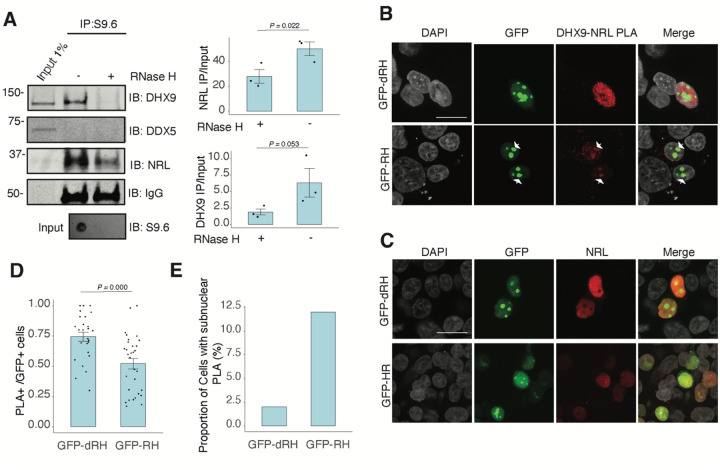
Nuclear R-loops regulate the interaction between NRL and DHX9. A) Western blot of DNA:RNA hybrid immunoprecipitation (DRIP) from adult mouse retina showing immunoblot (IB) staining for DHX9, NRL, and DDX5. Retinal genomic DNA (gDNA) was digested with MseI, DdeI, Alul, MboI, incubated with RNase III with/without RNase H and immunoprecipitated with S9.6 antibody/protein G beads. Retinal nuclear lysates were incubated with antibody/bead complexes. Quantification of signal intensities of immunoprecipitated DHX9 and NRL compared to input (n = 3). Data are presented as the mean ± SEM. Unpaired one-tailed t test was performed to compare means of samples against controls. B) Confocal image of HEK293 cells transfected with NRL and wild type (WT) human RNase H1 or D201N catalytic dead mutant EGFP fusions (GFP-HR, and GFP-dHR, respectively). PLA signals using antibodies for NRL and DHX9 are shown in red. Some cells displayed nucleolar-like accumulation of PLA signal (arrows). C) Confocal image of HEK293 cells transfected with NRL and GFP-dRH or GFP-RH and stained with antibodies against NRL (red). Nuclei are stained with DAPI (grey). Scale bar is 20 μM. D) Quantification of cells with positive PLA signals from B. Each dot represents a ratio of number of GFP+ cells with nuclear PLA signals per image. Data are presented as the mean ± SEM. Unpaired two-tailed t test was performed to compare means of samples against controls. E). Bar graph showing percentage of EGFP+ cells harboring NRL-DHX9 PLA signals in subnuclear compartments from B. Cells were counted in four independent assays (n = 83 and 85 cells for GFP-dHR and GFP-RH, respectively).

To assess whether NRL and DHX9 bind to retinal R-loops, we isolated R-loops from enzymatically digested retina gDNA and performed DNA-RNA immunoprecipitation (DRIP) using the S9.6 antibody. After incubations with mouse retinal nuclear lysates, NRL is enriched in R-loops along with DHX9 (Fig. 5A). However, DDX5 does not appear to bind to retinal R-loops, suggesting that DDX5 association with R-loops is weaker, transient, or more regulated in the retina, and in agreement with co-IP findings from RNase H treated bovine retina lysates. We then overexpressed GFP-tagged RNase H1 in HEK293 cells to reduce the R-loop levels, as shown previously^46^, and co-transfected the cells with human NRL. We detect a reduction in the number of cells displaying NRL-DHX9 nuclear PLA signals in cells that also harbor wild-type nuclear RNase H1 as compared to the cells having catalytically inactive version of RNase H1 (GFP-dHR) (Fig. 5B, D). In addition, we observe a small percentage of cells with PLA signals enriched in subnuclear compartments, especially in cells with wild-type RNase H1 (Fig. 5B, E, arrows). Notably, the localization of NRL or DHX9 is not influenced by the expression of RNase H1 (Fig. 5C, Fig. S4C). Thus, NRL and DHX9 interaction in the nucleoplasm is favored by R-loops, and R-loops appear to influence the stabilization and localization of NRL-DHX9 complexes.

### R-loops are enriched in neuronal genes and display distinct epigenetic signatures

**Figure 6.**
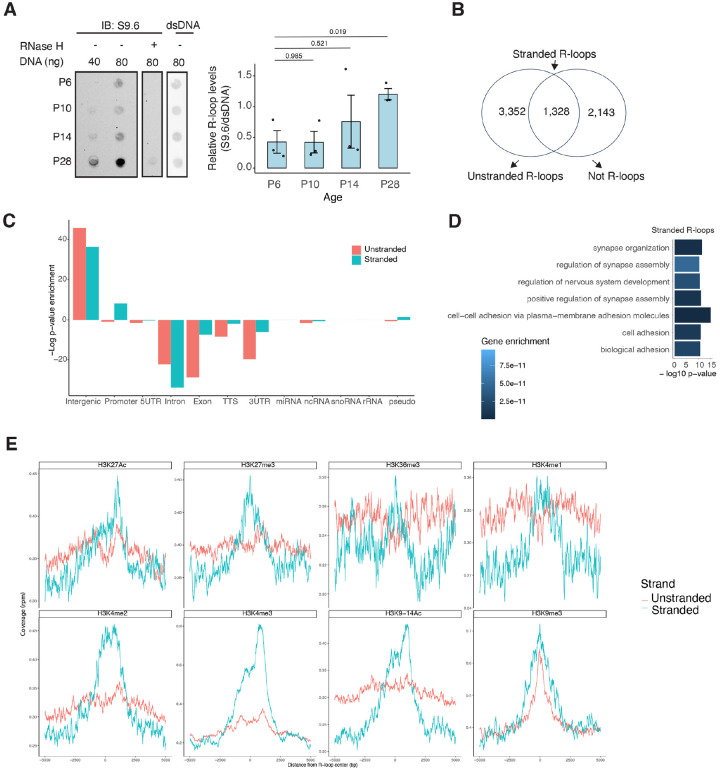
R-loops are dynamic in the mouse retina and associate with distinct epigenetic signatures. A) Dot blot of DNA:RNA hybrids from retinal gDNA. Retinas were dissected from mice at different developmental stages as shown. Genomic DNA (gDNA) was treated with RNaseIII with and without RNase H overnight. R-loops were detected using S9.6 antibody (n = 3). Data are presented as the mean ± SEM. Unpaired two-tailed t test was performed to compare means of samples against controls. B) R-loop peaks from ssDRIP-Seq were identified with narrow and broad peak parameters using RNase H treated samples (right circle) or opposite strand (left circle) for enrichment. The Venn diagram shows the R-loops kept for downstream analysis. R-loops found in at least 3 samples with a q-value < 0.001 in the narrow call or in the broad call were merged. C) Enrichment of unstranded and stranded R-loops at different genomic regions. D) Biological process enrichment of genes associated with stranded R-loops. E) Metaplot of H3K27ac, H3K27me3, H3K36me3, H3K4me1, H3K4me2, H3K4me3, H3K9–14ac, H3K9me3 signals centered on stranded and unstranded R-loop peaks.

NRL’s interplay with R-loops and R-loop proteins suggests a yet unexplored role of R-loops in regulating retinal transcriptional programs. Quantification of R-loop levels using the S9.6 antibody reveals an increase in R-loops with postnatal retinal maturation (Fig. 6A). This is in contrast with the relatively stable levels of total expressed genes (~15,000 > 1 count per million (CPM)) and transcripts (~ 35,000 > 1 RPKM (Reads Per Kilobase per Million mapped reads)) during retinal development^47^. Total R-loop levels increase in the *Nrl* KO retina, indicating a role of R-loops in controlling cell type-specific gene expression and/or the role of NRL in regulating R-loop levels (Fig. S5).

To identify genomic elements associated with R-loops, we performed single-strand DRIP (ssDRIP) followed by sequencing with four independent adult mouse retina samples and used corresponding RNAse H-treated controls. Remarkably, all four samples and all four RNAse-H treated samples clustered separately by their PC1 value (Fig. S6A, PCA), demonstrating the specificity of the assay. We identified either 4,000 or 2000 R-loops that were present in at least three samples, using a narrow or a broad peak caller, respectively. Most R-loops overlapped among these two peak callers, with a total of 4,677 peaks that were then used for further analysis (Figs. 6B and S6A). We also performed a stranded analysis, whereby we called peaks based on enrichment in reference to the opposite strand. This analysis generated 3,471 peaks, from which, we retained 1,328 stranded R-loops that were also seen during peak calling using RNase H as reference (Figs. 6B and S6B). Notably, the coverage was similar for both unstranded and stranded R-loops (Fig. S6C). R-loops identified intersected with different intergenic and genic regions and were overrepresented in intergenic and promoter regions (Fig. 6C). Intriguingly, unstranded R-loops were only enriched at intergenic regions (Fig. 6C). In agreement, stranded R-loops are preferentially observed around the TSS while unstranded R-loops are preferentially observed further downstream of the termination site (Fig. S6D).

We then studied whether certain genes/pathways display more R-loops in the retina. We found that stranded R-loops were particularly enriched at neuronal genes associated with synapse function (Fig. 6D) and unstranded R-loops were also enriched at genes associated with G protein-coupled receptor signaling (Fig. S6E). In addition, R-loops were identified in 20 genes involved in retinal disease including *Ush2a*, *Pcdh15*, and *Abcc6* (Table S4).

**Figure 7.**
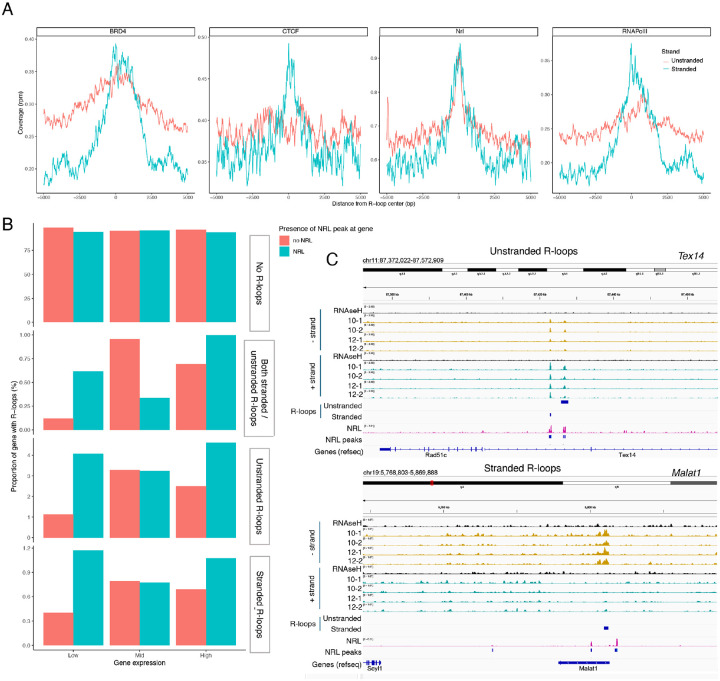
NRL is associated with different types of R-loops. A) Metaplot of BRD4 (SRR4252658), CTCF (SRR4252685), NRL (Cut&Run), and RNA pol II (SRR4252922) signals centered on stranded and unstranded R-loop peaks. B) Proportion of genes with and without stranded and unstranded R-loops and harboring NRL Cut&Run and Chip-Seq peaks. Genes were divided into low, mid and high according to their expression level. C) Genome view of *Text14* and *Malat1* mouse genes displaying ssDRIP-Seq signal in four retinas. Signals are shown for the positive (blue) and negative (orange) strands separately. RNase H treated samples are pooled and shown for each strand. Peak calls for NRL and unstranded and stranded R-loops are shown in blue.

Subsequently, we examined the association of R-loops with different chromatin states using published retina datasets^12^ (Fig. 6E). We observed that stranded and unstranded R-loops displayed differences in their epigenetic signatures. Stranded R-loops harbor histone marks associated with active transcription including H3K36me3, H3K4me2, and H3k4me3. However, unstranded R-loops were only enriched with H3K9me3, associated with heterochromatin (Figure 6E). Next, we investigated whether NRL binds to R-loops and found that NRL is enriched at both stranded an unstranded R-loops, suggesting a strong association of NRL to R-loops regardless of the epigenetic context (Fig. 7A). Notably, other chromatin binding factors such as BRD4, CTCF and Pol II were primarily associated with stranded R-loops. In addition, NRL binding was associated with a higher proportion of R-loops at low and high expression genes (Fig. 7B, Fig. S7A). Select examples of genes displaying NRL occupancy and harboring stranded or unstranded R-loops are shown in Fig. 7C. R-loops can also overlap promoter proximal or intergenic regions that do not harbor NRL binding (Fig. S7B). Taken together, these data indicate that R-loops are highly regulated in the retina and may exert gene-specific regulatory roles mediated by cell type-specific actors such as NRL. A model

## Discussion

RBPs are part of numerous nucleoprotein complexes that participate in diverse cellular processes including transcription of genes in appropriate spatiotemporal context^48,49^. Several RBPs interact with chromatin and function as integrators of transcription and co-transcriptional RNA processing^50^. We currently have poor understanding of how ubiquitously expressed RBPs are recruited to specific genomic loci at defined locations and in distinct cell types. Here, we demonstrate the interaction of retinal rod photoreceptor-specific bZIP transcription factor NRL with multitude of RBPs that are associated with RNA splicing as well as resolution of R-loops, which are obligatory during the transcription process. Focusing specifically on two helicases – DHX9 and DDX5, we show that their interaction with NRL is influenced by R-loops. Notably, R-loops are not uniformly present in genes that are highly expressed in the retina but are instead detected in genes associated with neuronal synapses and at heterochromatin regions. Our results suggest specific resolution and regulation of R-loops and provide insights into potential mechanisms by which RBPs and R-loops control transcriptional state in the retina.

Most R-loops are formed during transcription and play key roles in transcriptional initiation, elongation and termination. R-loops are reportedly sufficient to initiate transcription of antisense lncRNA from enhancers and other genomic regions^51^. R-loops also contribute to Pol II pausing at promoters and in gene bodies, thereby influencing transcriptional outputs^52^. Accumulation of R-loops at polyadenylation-dependent termination regions^53^ and R-loop-prone sequences downstream from poly A cleavage sites can cause transcriptional termination^54^. Additionally, R-loops may indirectly control gene expression by influencing chromatin structure and the epigenome^55^. In this study, we detect enrichment of R-loops at promoter and intergenic regions indicating their potential role in the modulation of distal and internal regulatory elements. We also demonstrate the presence of R-loops in genes irrespective of their expression levels and are specifically depleted from genes, such as rhodopsin, with high level expression and a high turnover in the retina. Our results suggest a limited formation or rapid removal of R-loops from such high-expressed genes, probably because of their requirement in maintaining retinal function and homeostasis.

We are intrigued by strong detection of R-loops in neuronal genes associated with synaptic structure and function, including those involved in cell-cell communication. As R-loops are generated during transcription, their relatively stable detection at genes encoding several neuronal and retina-disease associated proteins indicates distinct transcriptional control mechanisms for different gene families. One plausible explanation is slower turn-over of RNA and requirement for smaller protein amounts. Further investigations into R-loop formation and resolution at distinct genomic regions can offer mechanistic insights into non-coding variations relevant to disease.

Intriguingly, R-loops are also reportedly involved in maintenance of heterochromatin and telomers^56,57^. Our findings indicate that unstranded R-loops predominantly occur in intergenic regions and are associated with the heterochromatin mark H3K9me3. Thus, R-loops may play a crucial role in maintaining repressed chromatin stages in the retina, warranting further investigation.

NRL regulates the transcriptional state of many rod-specific genes^26^. The absolute requirement of NRL for rod photoreceptor cell fate determination and it’s continued high expression for functional maintenance of rods strongly indicate a multitude of roles of NRL beyond transcription initiation. Interactions of NRL with RBPs and R-loops are likely significant contributors to events during gene regulation. In particular, we note that NRL-interacting RNA helicases, DHX9 and DDX5, have a direct role in controlling R-loop resolution and cell fate specification^41^, DNA damage response^58^ and splicing regulation^59^. The interaction of NRL with DHX9 is very robust in GST pull downs, in HEK293 cells, and in bovine retina, suggesting that NRL-DHX9 complexes might play key roles in rod photoreceptors. Curiously, the interaction of NRL with DDX5 is weaker, suggesting that it may be more regulated or context dependent. In agreement, DHX9 but not DDX5 is enriched in retinal R-loops. Removal of RNA in bovine retina did not weaken the interaction between NRL and both DHX9 and DDX5, indicating that RNA is not required for many NRL-RBP interactions. Thus, our studies provide an avenue to investigate the function of R-loops in retinal development and function.

Excess of R-loops can generate DNA damage, genomic instability, and directly activate the immune response^46,60^. Dysregulation of R-loops is implicated in a variety of diseases including neurodegeneration^61^. Even during normal aging, aberrant transcription contributes to mis-splicing and increased R-loop formation in *Drosophila* photoreceptors^62^. As R-loops are low or absent at many key retina genes, failure of the mechanism that maintains these genes free of R-loops could contribute to retinal disease. Interestingly, monoallelic rare *DHX9* variants increase R-loop levels and are shown to be associated with neurological disorders^63^. Therefore, further investigation into NRL-DHX9 interaction with R-loops in the retinal photoreceptors could uncover mechanisms that maintain transcriptional homeostasis and genomic stability.

Transcription factors are reported to be involved in controlling alternate splicing^7^. Based on our discovery of NRL’s interaction with splicing factors and R-loop binding proteins, we hypothesize a potential role of NRL in determining patterns and rates of splicing, which is prevalent in photoreceptors^64–68^. NRL-RBP interactions may also influence the rate of transcription elongation and termination, and consequently impact transcript isoform diversity in rod photoreceptors. Notably, mutations in splicing factors as well as aberrant splicing of many genes are associated with retinal degeneration^65,69,70^.

**Figure 8.**
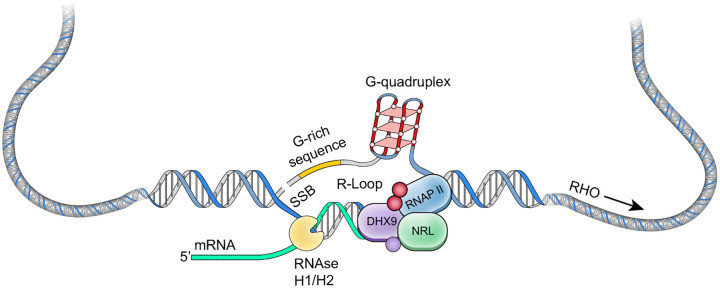
A putative model of R-loop regulation in retinal rod photoreceptors. We hypothesize that NRL guides R-loop resolvases such as DHX9 at photoreceptor genes to facilitate rapid expression of highly expressed genes (such as rhodopsin, RHO) and maintain genome integrity. SSB, single strand break.

Finally, our results indicate that other RNA-dependent chromatin processes may be influenced by NRL-RBP complexes. Many of the identified RBPs participate in maintaining chromatin architecture and modifications and in DNA damage repair, and their functions require further exploration. Overall, we propose that RBPs play key roles in rod photoreceptors through their interactions with NRL. A proposed model for R-loop regulation in photoreceptors is presented in Fig. 8. Future studies will aim at unravelling the physiological significance of NRL-interacting RBPs on R-loops and chromatin regulatory mechanisms in photoreceptors.

## Methods

### Mouse strains and husbandry

All procedures involving mice were approved by the Animal Care and Use Committee of the National Eye Institute (NEI-ASP#650). C57BL/6J (B6) mice were kept in a 12 hr light/12 hr dark cycle and fed ad libitum at the NEI animal facility. Both male and female mice were used in this study.

### Human tissue

Human donor eyes were procured from Lions World Vision Institute (Tampa, FL). Autopsy material from unidentified deceased individuals is not subject to Institutional Review Board (IRB) review and does not need a determination from the Office of Human Subjects Research Protections (OHSRP) according to 45 CFR 46 and NIH policy (OHSRP ID#: 18-NEI-00619). Eyes were enucleated within 6 hr of death, followed by making a 2 cm incision at the limbus before immersing in a solution of 4% formaldehyde in PBS. Eyes were subsequently transferred to PBS for shipment and kept at 4°C. Before sectioning, eyes were fixed in 4% formaldehyde in PBS for 2 hr and immersed in 30% sucrose.

### Antibodies

The following antibodies were used: DDX5 Monoclonal antibody (Cat. No. 67025-1-Ig), DHX9 Polyclonal antibody (Cat. No. 17721-1-AP), and HNRNPA2B1 (Cat. No. 14813-1-AP), all obtained from Proteintech (Rosemont, IL, USA); HNRNPA1 (Cat.No. 8443, Cell Signaling, Danvers, MA, USA); DNA-RNA Hybrid antibody S9.6 (Cat. No. 65683, Active Motif, Carlsbad, CA, USA); Xpress Monoclonal Antibody (Cat. No. R910-25, ThermoFisher, Waltham, MA, USA); HNRNPM (Cat. No. A6937, Abclonal, Woburn, MA, USA); HNRNPU and HNRNPUl1 (Cat. No. MA1-24632 and Cat. No. 10578-1-AP, respectively, ThermoFisher, Waltham, MA); rabbit anti-NRL (Swaroop lab^27^ and R&D systems Cat. AF2945); double stranded DNA (Cat. No. MAB030, MilliporeSigma, Burlington, MA, USA).

### Plasmids

Human NRL constructs in pcDNA^™^4/HisMax vector containing an N-terminal Xpress tag (Cat. No. V86420, ThermoFisher Scientific, Waltham, MA, USA) or in pGEX-4 T2 plasmid containing an N-terminal GST tag (GE Healthcare/Cytiva; cytivalifesciences.com) have been described previously^71^. Human RNase H1 or D201N catalytic dead mutant EGFP fusions (GFP-HR, and GFP-dHR) were developed in the Cimprich lab^46^ and obtained from Addgene (Cat. No.196702, and 196703).

### Mass spectrometry

Affinity-purified protein samples after incubation of GST-NRL (n = 3 for GST-NRL; n = 3 for GST only control) with bovine (n = 2 for NRL immunoprecipitation, IP; n = 1 for IgG control) or mouse retina (n = 2 for NRL IP, n = 1 for IgG control) were subjected to Liquid Chromatography by Tandem Mass Spectrometry (LC-MS-MS) analysis (Poochon Scientific, Frederick, MD, USA). Samples were digested with trypsin, peptides were extracted, desalted and analyzed using Q-Exactive hybrid Quadrupole-Orbitrap Mass Spectrometer and Thermo Dionex UltiMate 3000 RSLCnano System (ThermoFisher Scientific, Waltham, MA, USA). Peptides were ionized and sprayed into the mass spectrometer using Nanospray Flex Ion Source ES071 (Thermo Scientific, Waltham, MA, USA). Raw data files were searched against bovine, mouse or human protein sequence databases (National Center for Biomedical Information, NCBI) using Proteome Discoverer 1.4 software (Thermo Scientific, Waltham, MA, USA) based on SEQUEST algorithm. Carbamidomethylation of cysteines was set as a fixed modification, whereas oxidation and deamidation Q/N-deamidated (+0.98402 Da) were set as dynamic modifications. The minimum peptide length was specified to be five amino acids. The precursor mass tolerance was set to 15 ppm and fragment mass tolerance to 0.05 Da. The maximum false peptide discovery rate was specified as 0.01. All assembled proteins with peptides sequences and matched spectrum counts (peptide spectrum match counts (#PSM)) were included in the analysis.

### Yeast two-hybrid (Y2H) screening

We performed Y2H assays using a modified Matchmaker System (Clonetech, Mountain View, CA, USA) with a human retinal cDNA prey library pGADT7 vector (containing Gal4 activating Domain). Two partial NRL domains were synthesized by Genewiz (Germatown, MD, USA): (1) NRL Extended Homology Domain (EHD) with the Basic Motif (BM) (residues 95–175), and (2) partial NRL BM with NRL bZIP domain (residues 176–237). NRL domain containing inserts were subcloned into the bait vector, pGBKT7 (containing Gal4 binding domain), and transformed into Y2HGold yeast. The yeast containing a bait were inoculated at 30°C overnight in 50 mL (−)Trp broth to obtain a cell yield of 7.5×10^8^ cells per culture. Subsequently, yeast cells were pelleted at 3000×g for 5 min, resuspended in 50 mL of (−)Trp broth and incubated at 30°C for 3–4 hr with shaking (225 rpm). Yeast colonies containing baits were transformed with 10 μg of human retinal prey cDNA library. For screening, positive colonies were selected on SD/-Trp/-Leu/X-alpha-gal/Aureobasidin-A (DDO/X/A) media to determine β-galactosidase activity and antibiotic resistance as an indicator of a positive interaction with NRL. These interactors were validated by patch plating onto higher stringency SD/-Trp/-Leu/-Ade/-His/X-alpha-gal/Aureobasidin-A (QDO/X/A) media. Positive interactor inserts were PCR amplified, sequenced using T7 primer, and identified by Blastn and Blastx (NCBI).

To confirm NRL interacting RBPs, colonies containing RBP prey and NRL domain baits were resuspended in ultra-pure water, and plated against controls on 5 different plating media: SD/-Trp, SD/-Leu, SD/-Trp/-Leu, SD/-Trp/-Leu/X-alpha-gal/Aureobasidin-A and SD/-Trp/-Leu/-Ade/-His/X-alpha-gal/Aureobasidin-A.

### Cell culture and transfection

HEK293 cells were cultured in Dulbecco’s modified Eagles’s medium (Cat. No. 11885084, ThermoFisher Scientific, Grand Island, NY, USA) Containing 10% fetal calf serum (Cat. No. S11550, R&D Systems, Flowery Branch, GA, USA), 100-units/mL penicillinG and 100 μg/mL streptomycin (Cat. No. 15140122, ThermoFisher Scientific, Grand Island, NY, USA). Cell transfection was performed with X-tremeGENE 9 DNA Transfection Reagent (Cat. No. 6365779001, Cat. No. 06365787001, Roche, Mannheim, Germany) or Lipofectamine 2000 (Cat. No. 11668027, ThermoFisher Scientific, V Carlsbad, CA, USA) per manufacturer’s instructions.

### Immunofluorescence and microscopy

Eyes from *Nrl* WT or Nrl-knockout (KO) C57BL/6 mice^18^ at postnatal day (P) 28 were fixed in 4% paraformaldehyde for 1 hr, washed 3x in PBS, and embedded in optimal cutting temperature (OCT) medium on dry ice. For NRL detection and proximity ligation assay (PLA), eyes were directly embedded in OCT. Human eyes were fixed for 2 hr in 4% PFA, washed as above and embedded in OCT. Eyes were cryosectioned at 14 μm and mounted on SuperFrost Plus slides (Thermo Fisher Scientific, Waltham, MA, USA). For NRL detection and PLA in mouse retinas, fresh sections were postfixed in 4% PFA for 7 min. Retinal sections were then incubated in blocking solution (5% bovine serum albumin, 0.3% Triton X-100 in PBS) for 1 hr followed by primary antibody (anti-DDX5, anti-DHX9 or anti-NRL 1:100) incubation in blocking solution at 4°C overnight. After three washes of 10 min each in PBS, sections were incubated in secondary antibody in blocking solution in the presence of the DNA dye DAPI (4′,6-diamidino-2-phenylindole) for 1 hr. Sections were washed 3x in PBS and mounted in ProLong^®^ Gold Antifade Reagent (Life Technologies Inc., Carlsbad, CA, USA). Confocal images were acquired using SP8 Leica 2-photon confocal microscope or a Zeiss LSM 800 point scanning confocal microscope using the AiryScan detector.

### Proximity ligation assay (PLA)

PLA was carried out using Duolink^®^ PLA Fluorescence kit (Millipore, Sigma) per manufacturer instructions. Briefly, HEK293 cells were transfected with NRL-Xpress or RNase H1 constructs (GFP-HR and GFP-dHR). Cells were washed with PBS 48 hr post-transfection, fixed using 4% paraformaldehyde in PBS for 15 min at room temperature and washed three times with PBS. Mouse and human retinas were fixed as described above. Cells or sections were permeabilized using 0.3% Triton X in PBS for 5 min. Duolink blocking solution was added for 60 min at 37°C. Rabbit anti-DHX9, anti-DDX5 antibodies, anti-NRL or Xpress antibodies were diluted in Duolink antibody diluent and incubated overnight at 4°C. After washing, plus and minus PLA probes were diluted 1:5 into Duolink antibody diluent. Cells were incubated at 37°C for 1 hr. After further washing, the ligation was performed for 30 min at 37°C.

### Protein lysates and enzymatic treatments

#### Bovine Sephacryl S-300 fractions.

High molecular mass complexes containing NRL were obtained by size exclusion chromatography as described earlier^71^. Briefly, bovine retinal nuclear extracts were fractionated using 40% ammonium sulfate. Isolation of high molecular mass protein complexes was performed using HiPrep 16/60 Sephacryl S-300 High-Resolution column (GE Lifesciences/Cytiva). The peaks corresponding to 650 and 450 kDa containing NRL were immunoprecipitated using NRL antibody/protein A bead complexes overnight at 4°C, as described earlier.

#### Bovine nuclear extracts for GST purification.

Retinas were resuspended in CE1 buffer (10 mM HEPES-KOH pH 7.5, 1.5 mM MgCl_2_, 10 mM KCl, 10% Glycerol, 1 mM DTT) with protease inhibitors (PI) for 10 min at 4°C. Retinas were homogenized in dounce homogenizer 25 times and pelleted at 250×g for 10 min. Nuclear pellets were resuspended in high salt buffer (450 mM NaCl, 10 mM Tris pH 7.4, 2 mM EGTA, 0.1% Triton X-100, 2 mM MgCl_2_) with PI for 30 min at 4°C. Chromatin was pelleted at 10,000×g for 10 min. Supernatants were diluted to 150 mM NaCl and incubated with BSA-blocked antibody-bead conjugates overnight as described earlier. For RNase A treatments, lysates were incubated with RNase A at 1 μg per μg DNA for 30 min at 37°C before antibody incubation.

#### Bovine and HEK293 nuclear extracts and treatments.

HEK293 cells cultured in 6-well plates as described earlier, were lysed in 1 mL co-immunoprecipitation (co-IP) binding buffer (40 mM Tris-HCl pH 8.0, 150 mM NaCl, 2 mM EDTA and 0.2% NP-40) containing protease inhibitor cocktail. The nuclear pellet from bovine retina was obtained as described above and resuspended in 1 ml of co-IP binding buffer. After sonication using an ultrasonic liquid processor (MISONIX, Inc, Farmingdale, NY) until complete chromatin dissolution, samples were clarified by centrifugation at 16,000×g for 10 min at 4°C. The DNA concentration in the lysate was measured by Qubit dsDNA BR Assay Kit (Thermo Fisher Scientific, Waltham, MA). For each immunoprecipitation, 100 μl lysate was treated as follows: For control, the lysate was mixed with 50 units of RNaseOUT recombinant ribonuclease inhibitor (Thermo Fisher Scientific, Waltham, MA, USA); for RNAse A treatment, the lysate was mixed with 5 μg of RNase A per μg of DNA (Thermo Fisher Scientific, Waltham, MA, USA); for RNase H treatment, 2.5 units of RNase H were added per μg of DNA with 1x RNase H buffer (New England Biolabs, Ipswich, MA); for DNase I treatment, 1 unit of DNase I was added per μg of DNA with 1x DNase I reaction buffer (Thermo Fisher Scientific, Waltham, MA). All reactions were performed for 30 min at 37°C. After treatments, the lysates were incubated with antibodies as described above.

### Co-immunoprecipitation (co-IP)

Dynabeads Protein A (Thermo Fisher Scientific, Waltham, MA) were blocked with 1% BSA at room temperature for 1 h and incubated with 1 μg of the respective antibodies for another hour. After washing off the unbound antibody, the lysates were added to the beads and incubated overnight at 4°C. The beads were then washed 3 times for 15 min each with co-immunoprecipitation wash buffer (150 mM NaCl, 10 mM Tris-HCl pH 7.4, 2 mM EGTA, 1% Triton X-100, 2 mM MgCl_2_) and bound protein complexes were eluted by boiling in Laemmli SDS sample buffer for 10 min at 95°C.

### GST affinity purification

GST-NRL was expressed in BL21(DE3) competent cells (ThermoFisher Scientific, Waltham, MA) using 0.2 mM IPTG for 16 hr at 20°C and purified using Glutathione Sepharose beads (Cat. No. GE17-0756-01, MilliporeSigma, Burlington, MA) by incubating BSA-blocked beads with cell lysates for 3 hr at 4°C. GST-NRL beads were then incubated for 2 hr at 4°C with nuclear fraction from bovine retina (prepared as above) with or without 25 U of benzonase. Afterwards, the beads were washed three times for 10 min each in PBS with 1% Triton X-100, then boiled in Laemmli sample buffer containing 100 mM DTT for 7 min and stored at −80°C or loaded into 4–15% acrylamide gels for immunoblotting.

### R-loop detection

R-loops were extracted as previously reported^72^ with some modifications. Retinas or HEK293 cells were incubated in nuclear extraction buffer (Tris pH 7.4 10 mM, NaCl 10 mM, MgCl_2_ 3 mM, BSA 1%, Tween-20 0.1 %, NP-40 0.1%) for 10 min. After dounce homogenization, nuclei were pelleted at 500×g for 5 min at 4°C. Genomic DNA was obtained by incubating nuclei in 10 mM Tris-HCl containing 1 mM EDTA (TE) with 1% SDS and 60 μg Proteinase K20 overnight at 37°C. DNA was extracted with Phenol/Chloroform Isoamyl alcohol 25:24:1 (PCI). Briefly, equal volume of PCI was added to each sample. After gentle mixing, samples were spun down at 12,000×g for 30 min at 4°C. DNA was precipitated by adding 1/10 volume of 3 M NaOAc, pH 5.2 and 2.5 volumes of 100% (vol/vol) ethanol. The pellet was washed with 1 mL of 70% ethanol. After gentle mixing, samples were centrifuged at 12,000×g for 15 min at 4°C. The supernatant was discarded, and the pellet was air dried and resuspended in TE buffer. DNA was incubated with RNase H buffer and RNase III at 37°C OV with and without RNase H. To detect R-loops during development, retinas were collected from three B6 mice at each time point (postnatal day (P)6, P10, P14 and P28).

#### Dot blot analysis.

gDNA samples diluted in TE buffer were blotted on a positively charged Hybond ^®^ -N+ hybridization membrane (Thermo Fisher Scientific, Waltham, MA, USA) using a dot-blot apparatus (Cat. No. 1706545, Bio-Rad Laboratories, Hercules, CA, USA). Membranes were crosslinked using 1200 μJ UV light.

### Electrophoretic Mobility Shift Assay (EMSA)

DNA oligonucleotides (5 pmoles, forward strand) were incubated with 50 μCi of ^32^P-ATP g and 10 units of T4 polynucleotide kinase for 30 min at 37°C. The reaction was terminated by heating at 65°C for 10 min and column purified to remove unincorporated label. This labeled oligonucleotide was annealed to 5 pmoles of the complementary strand by heating to 95°C for 5 min and cooling slowly to room temperature. Double stranded ^32^P-labeled oligonucleotides (0.2 pmol, 20,000 cpm) were incubated with 5 μg of bovine nuclear and 1X Binding Buffer (LightShift, Thermo Fisher Scientific), 50 ng/μl Poly dI-dC, and 5 mm MgCl_2_ for 1 hr. Reactions were terminated by addition of loading buffer, separated on 8% DNA retardation gel (Invitrogen, Waltham, MA, USA). Subsequently, the gel was dried and exposed to Phosphor screen overnight, which was scanned using Typhoon FLA 9500 Biomolecular Imager (GE Lifesciences). Cold competition assays were performed by preincubating unlabeled double stranded oligonucleotides at 0.1 to 0.2 pmole concentration, as indicated, with the nuclear extract for 5 min before the addition of the labeled probes. Mutant probes were incubated at 0.2 pmole.

Probes for EMSA were designed by examining putative NRL binding motifs in ATAC-Seq footprints overlapping with NRL ChIP-Seq peaks as described^45^.

Probe for DHX9: 5’ GAGGTTGCTGAGCCCCGCCCCCTC 3’.

Mutant DHX9 probe 1: 5’ GAGGTCAATTTGCCCCGCATTCTC 3’.

Mutant DHX9 probe 2: 5’ GAGGTACCCTTGCCCTTCAACCTC 3’.

### Protein Network

RBPs included in the analysis had >2-fold increase compared to the controls and were present at 10 or more total peptide spectrum matches (PSMs) in experimental samples. Protein-protein interactions (PPI) among RBPs were extracted from STRING Database (https://string-db.org/). Only data identified experimentally was included for analysis. The confidence threshold was set to 0.4 and displayed in the network as edge thickness. Networks were created using Cytoscape (https://cytoscape.org/).

### Gene regulation analysis

Normalized transcripts per million (nTPM) of neuronal cells from single-cell human data (https://www.proteinatlas.org/) was used to compare expression levels in photoreceptors and other neuronal cells. Overlaps between genes encoding RBPs (+/−1 kb from gene body) identified in two out of four assays with human retina NRL-ChIP-Seq peaks and super-enhancers from human retina (obtained from^45^) were identified using bedtools (https://bedtools.readthedocs.io/en/latest/). Genomic views of gene loci were obtained using IGV (https://software.broadinstitute.org/software/igv/).

### S9.6 DNA-RNA co-immunoprecipitation

DRIP was performed as described^41,73^, with minor modifications. Genomic DNA was digested with 50 U of Mse I, Dde I, Alu I, and Mbo I. Digested DNA (500 ng) was treated with RNase III and/or RNase H overnight as above. gDNA was added to Protein G Dynabeads (Thermo scientific) preincubated with 1.5 μg of S9.6 antibody for 1 hr at RT in 500 μl of binding buffer (10 mM Na2HPO4, 140 mM NaCl, 0.05% Triton X-100). After three washes in binding buffer, R-loop-antibody-protein G complex was incubated with mouse retina lysate (200 μg) overnight at 4°C. Nuclear lysates were obtained as described above and pretreated with RNase A (0.1 ng per 1 ug DNA) for 1 hr at 37°C followed by incubation with 1000 U RNaseOut for 10 min at 4°C. After 3 washes with IP buffer, S9.6-bound complexes were analyzed by immunoblotting.

### Single-stranded DNA-RNA immunoprecipitation (ssDRIP)-Seq

ssDRIP-seq was performed as described earlier^41^. The libraries were prepared using the Accel-NGS 1S Plus DNA Library Kit (IDT technologies Coralville, IA, USA). Briefly, the DRIPed DNA sample obtained as above was fragmented to an average length of 250 base pairs (bp) utilizing an S220 Focused-ultrasonicator (Covaris, Woburn, MA, USA). The fragmented DNA was then denatured by heating at 98°C for 2 min, followed by immediate cooling on ice for 2 min. The R2 adapter was first ligated to the 3′ end of the ssDNA, followed by an extension step. Subsequently, the R1 adapter was ligated to the 5′ end. After PCR amplification, the resulting libraries were purified using AMPure XP beads (Beckman Coulter, San Jose, CA). The quality of each library was assessed with a TapeStation instrument (Agilent, Palo Alto, CA, USA), and sequencing was carried out on an Illumina NextSeq 2000 system (San Diego, CA, USA) at 2 × 100 bp at a final library concentration of 1100pM with a sequencing dept of grater that 20 million pair end reads.

After trimming adaptor sequences, low-complexity tails were removed by further trimming 10 bps from each read on the 3’ end of the first read in the pair and 5’ end of the second read in the pair. Reads were aligned to the mouse genome (mm10) using Bowtie2. Duplicated reads, reads with score < 20 (including multi-mapping reads) and reads mapping ENCODE consortium blacklisted regions of mm10 genome (github.com/Boyle-Lab/Blacklist/) were removed. Mapped reads were split by strand using samtools. To confirm the quality of the experiment, a PCA was run on the 500bp coverage scaled in R for all samples treated or not with RNAse-H, using R ggfortify package. R-loops calling was performed using MACS2. First, all R-loops (without regard of any strand specificity) were called on all reads, using the merged RNase H treated samples as control dataset using MACS2 with the default parameters (narrow peaks) and the broad peaks option. R-loops found in at least 3 samples with a q-value ≤ 0.001 in the narrow call or in the broad call were merged. Then, strand specific enriched peaks (including strand-specific R-loops and non-specific signal) were called on reads split by strand, using the reads mapping the opposite strand as control dataset. We also used both MACS2 with the default parameters (narrow peaks) and the broad peaks option. Strand-specific peaks found in at least 3 samples with a q-value ≤ 0.001 in the narrow call or in the broad call were merged. Finally, from these two lists of peaks (total R-loops and strand-specific peaks), we selected the unstranded R-loops as the R-loops not found in the strand-specific peaks and the stranded R-loops as the peaks present in both the R-loops list and the strand-specific peak. R-loops annotation, gene ontology analysis and chromatin marks enrichment were computed using HOMER and plotted using R package ggplot2. Overlap between gene expression levels, R-loops and NRL peaks were computed using Bedtools. For this, we utilized our previously published expression data that was reanalyzed for GRCm38/ENSv98 annotation^47^, re-analyzed NRL chromatin binding data (ChIP-seq from ref.^25^ and Cut&Run from ref.^27^). We generated related plots using the R package ggplot2 and chromatin profiles using IGV.

### Statistical analysis

Means between groups were compared using Student’s t-test in at least three biological replicates. The differences were considered statistically significant with a two or one--tailed p-value of < 0.05. Immunoblots of pull-downs were analyzed using ImageJ (magej.net/ij/index.html) by calculating the ratio of signal intensities of prey and bait normalized to controls. Dot blot signal intensities were normalized to input gDNA. R-loop protein enrichment was calculated using the Fisher exact test with a contingency table based on the total number of retina-expressed nuclear RBPs. Mouse nuclear RBPs data were obtained from the RBP2GO database (https://rbp2go.dkfz.de/), which reports 1764 RBPs including 1534 that are expressed in the mouse retina.

## Data Availability

All sequencing data that support the findings of this study have been deposited in the National Center for Biotechnology Information Gene Expression Omnibus (GEO) and are accessible through the GEO Series accession number GSE274666. Go to https://www.ncbi.nlm.nih.gov/geo/query/acc.cgi?acc=GSE274666 Enter token oputuwwyjhsxdib into the box All other relevant data are available from the Lead Contact on request.
